# Weighted lambda superstrings applied to vaccine design

**DOI:** 10.1371/journal.pone.0211714

**Published:** 2019-02-08

**Authors:** Luis Martínez, Martin Milanič, Iker Malaina, Carmen Álvarez, Martín-Blas Pérez, Ildefonso M. de la Fuente

**Affiliations:** 1 Department of Mathematics, University of the Basque Country UPV/EHU, Bilbao, Spain; 2 Biocruces Bizkaia Health Research Institute, Barakaldo, Spain; 3 Basque Center for Applied Mathematics BCAM, Bilbao, Spain; 4 University of Primorska, UP IAM and UP FAMNIT, Koper, Slovenia; 5 IDIVAL Valdecilla Biomedical Research Institute, Santander, Spain; 6 Department of Nutrition, CEBAS-CSIC Institute, Murcia, Spain; UPMC, FRANCE

## Abstract

We generalize the notion of λ-superstrings, presented in a previous paper, to the notion of weighted λ-superstrings. This generalization entails an important improvement in the applications to vaccine designs, as it allows epitopes to be weighted by their immunogenicities. Motivated by these potential applications of constructing short weighted λ-superstrings to vaccine design, we approach this problem in two ways. First, we formalize the problem as a combinatorial optimization problem (in fact, as two polynomially equivalent problems) and develop an integer programming (IP) formulation for solving it optimally. Second, we describe a model that also takes into account good pairwise alignments of the obtained superstring with the input strings, and present a genetic algorithm that solves the problem approximately. We apply both algorithms to a set of 169 strings corresponding to the Nef protein taken from patiens infected with HIV-1. In the IP-based algorithm, we take the epitopes and the estimation of the immunogenicities from databases of experimental epitopes. In the genetic algorithm we take as candidate epitopes all 9-mers present in the 169 strings and estimate their immunogenicities using a public bioinformatics tool. Finally, we used several bioinformatic tools to evaluate the properties of the candidates generated by our method, which indicated that we can score high immunogenic λ-superstrings that at the same time present similar conformations to the Nef virus proteins.

## Introduction

Infectious and transmissible diseases cause deaths of millions of people every year. The best immunological measures to prevent such diseases are vaccines. Therefore, the main efforts of immunologists are focused towards improving our predictions of effective epitopes that would confer protection against pathogens [1] and towards enhancing our ability to select appropriate epitopes for inclusion in an efficient vaccine [2]. Protective immunity requires humoral or cellular immunity depending on the pathogen.

Humoral immunity implies the production of antibodies by B cells that interact with surface or secreted toxins of pathogens. Each antibody binds to an epitope, defined as the three-dimensional structure of amino acids that can be contacted by the variable region of an antibody. There are two types of B-cell epitopes: (i) linear or continuous epitopes, which are short peptides that correspond to a fragment of a protein, and (ii) conformational epitopes, composed of amino acids not contiguous in primary sequence of the protein but brought in close proximity within the folded 3D structure. The length of these epitopes is variable, ranging from 8 to 20 amino acids [3].

Cellular immunity depends on T-cell epitopes generated in other cell types, the antigen presenting cells (or APC) that generate linear epitopes from pathogen degradation or protein synthesis. These short linear amino acids generated from intracellular degraded or synthesized proteins from the microorganisms bind to two types of major histocompatibility complexes (MHC), class I MHC that attach epitopes of 8-9-mer lengths and class II MHC that fit epitopes of 12-15-mer lengths [4]. CD4+ T cells recognize class II MHC epitopes and CD8+ T cells recognize class I MHC epitopes in APC.

Bionformatics methods that predict B-cell epitopes are based on certain correlations between some physicochemical properties of amino acids and the locations of linear B-cell epitopes with protein sequences [5]. Therefore, hydrophilicity, flexibility, turns, and solvent accessibility generated propensity scales for B-cell epitope prediction. However, propensity scale predictions have failed to predict B-cell epitopes since they are mainly based on fixed lengths and require flexibility [6].

Mapping of T-cell epitopes has been based on using complete sets of overlapping peptides or biochemical elution methods from MHC molecules. Both methods, when applied to a classical T cell-mediated pathogen as *Listeria monocytogenes* were costly, time consuming and, more importantly, failed to generate predictive rules [7], [8]. More recently, bioinformatics methods have also been applied to T-cell epitopes via their ability to bind MHC molecules [9]. However, they have not been able to predict efficient epitopes for vaccine design. Therefore, a mathematical method of epitope prediction able to be applied either to B or T-cell epitopes is important in the immunology field of vaccination. This has been highlighted in the last outbreaks of world wide infectious diseases, such as flu every year or Ebola in the most recent years.

Martínez et al. [10] introduced the notion of a λ-*superstring* along with an optimization problem associated to it, and gave an application to the computational design of vaccines. Given two sets of strings, a set of *host strings*, which models a set of instances of a protein (which in our case will be amino acid sequences of the protein for a given pathogen), and a set of *target strings*, which models a set of epitopes, a λ-superstring was defined to be a string that models a candidate vaccine containing, as substrings, at least λ target strings from each host string. This means that the vaccine covers at least λ epitopes in each patient. The associated optimization problem was to find a λ-superstring of minimum length, which means to find a candidate vaccine as short as possible. The aforementioned problem in [10] was shown to generalize both the shortest common superstring problem and the set cover problem, and in order to solve it they gave two approaches, one to find exact solutions and the other one to obtain approximate solutions. The approach giving exact solutions was based on an integer programming formulation of the problem, under the assumption that no two target strings are comparable with respect to the substring relation.

Motivated by the necessity of selecting the most effective epitopes mentioned at the beginning of this section, we give in this paper a generalization of the notion of λ-superstring and of the corresponding optimization problem, which is more biologically meaningful. We consider a weight function for the target strings, which represents the immunogenicity of each epitope. A *weighted* λ-*superstring* is then defined as a string such that for every host string, the sum of the weights of all target strings covered simultaneously by the string and the host string is at least λ (i.e., the minimum of the sums of predicted immunogenicities of the epitopes in each protein variant considered is at least λ). Note that, in principle, the model allows for negative weights. On one hand, the more negative the immunogenicity of an epitope is, the less we prefer the corresponding target string to be a substring of a weighted λ-superstring. However, it could happen that a short weighted λ-superstring necessarily contains target strings representing epitopes of large positive immunogenicity (indicating that its epitopes will likely induce an immune response), which together cover a target string representing an epitope of negative immunogenicity (meaning that it is very unlikely for those covered epitopes to generate an immune response). Furthermore, in the Materials and Methods section we will present a model that also takes into account good pairwise alignments of the obtained superstring with the host strings, in which case target strings with negative weights could be essential. Therefore, we cannot simply disregard target strings with negative weights from the model.

We give two methods for obtaining short weighted λ-superstrings in the Materials and Methods Section. In the first subsection, a mathematical formulation of the problem is presented. In the second subsection, following the approach of [10], a graph theoretic formulation of the problem is given, from which an integer program is derived leading to optimal solutions to the problem of finding shortest weighted λ-superstrings. Next, in the third subsection, a genetic algorithm is introduced to obtain suboptimal solutions in the case when the integer programming approach cannot be used due to the large number of variables in the IP formulation. This algorithm, besides getting the λ-superstring criterion closer to biological reality, considers an additional objective to be optimized simultaneously, the alignment of the protein. By optimizing the alignment, we can obtain vaccine candidates that resemble the virus proteins that are recognized by the immune system, and therefore, build a pseudo-protein that will have a stable structure, recognizable by the MHC-complex. Our genetic algorithm is based on the NSGA-II algorithm [11], which is one of the most used heuristic techniques for solving multi-objective problems, which stands out due to its high speed, elitism, and non-necessity of specifying a sharing parameter for the optimization. In the Results section we give an application to the design of a weighted λ-superstring for a set of target strings corresponding to the Nef protein of HIV-1. We chose Nef because it is highly immunogenic [12] and plays an important role in HIV pathogenesis [13]. In order to evaluate the goodness of our candidate in silico, we have used several bioinformatic tools such as Blast, VaxiJen, I-Tasser and Phyre-2. In addition, we have studied the mismatch proportion, and compared our candidate to a candidate obtained by LANL’s Epigraph, a consensus sequence and to one of the solutions using the unweighted algorithm from [10]. Finally, in the Discussion section, the main conclusions are presented and some future lines of research are outlined.

## Materials and methods

### The shortest weighted λ-superstring problem

In this subsection, we give a mathematical formulation of the problem. We first recall some notation and terminology for finite strings (that is, finite sequences) over a finite alphabet *A*. We denote by *ϵ* the empty string, and by A* the set $A^{*}=\cup_{n=1}^{\infty }A^{n}\cup \left\{\epsilon \right\}$ of all finite strings over *A*. It is well known (and can be easily seen) that the set A* forms a semigroup with respect to the operation + of concatenation (*s*_1_, …, *s*_*n*_) + (*t*_1_, …, *t*_*m*_) = (*s*_1_, …, *s*_*n*_, *t*_1_, …, *t*_*m*_). Given a string **s** = (*s*_1_, …, *s_n_*) ∈ A*, we denote by *ℓ*(**s**) the *length* of **s**, that is, *n*. We say that a string **s** is a *substring* of another string **t**, and denote this relation by **s** ⊆ **t**, if **t** can be written as **t** = **u** + **s** + **v** for some strings **u** and **v** over *A*. We also use ⊂ to denote the proper substring relation, that is, **s** ⊂ **t** if and only if **s** ⊆ **t** and **s** ≠ **t**. Given two strings **s** = (*s*_1_, …, *s_n_*), **t** = (*t*_1_, …, *t_m_*) in A*, the *degree of overlapping* of **s** and **t** is defined as

$$\begin{array}{c} ov\left(s,t\right)=\max\{i\in \left\{0,1,\ldots ,\min\left\{m,n\right\}\right\}|s_{n-i+j}=t_{j}\;\text{for}\;j=1,\ldots ,i\}\;. \end{array}$$

The operation of the *overlapping sum*+′ in A* is defined by

$$\begin{array}{c} \left(s_{1},\ldots ,s_{n}\right){+}^{\prime }\left(t_{1},\ldots ,t_{m}\right)=\left(s_{1},\ldots ,s_{n-ov(s,t)}\right)+\left(t_{1},\ldots ,t_{m}\right). \end{array}$$

We remark that this operation is not associative.

The combinatorial approach to the design of vaccines described in [10] is based on the notions of λ-superstrings and λ-cover superstrings, which we now recall. Given two finite sets *H*, *T* ⊆ A* of *host* and *target* strings (modeling the set of instances of the chosen pathogen protein and the set of epitopes), respectively, and a positive integer λ, a λ-*superstring* for (*H*, *T*) is a string **v** ∈ A* such that for every host string **h** ∈ *H*, there exist at least λ strings in *T* that are common substrings of both **h** and **v**. Similarly, given a collection C of finitely many finite sets of strings over *A* (that is, $C=\{X_{1},\ldots ,X_{n}\}$ where *X*_*i*_ ⊆ A* for all *i* ∈ {1, …, *n*}) and a positive integer λ, a λ-*cover superstring* for C is a string **v** ∈ A* such that for every X∈C, at least λ strings in *X* are substrings of **v**.

We now generalize these notions and the corresponding optimization problems to the weighted case.

**Definition 1** *Let H, T* ⊆ A* *be two finite sets of host and target strings, respectively, let each target string* **t** ∈ *T be equipped with a weight* w(t)∈R, *and let* λ∈R. *A weighted* λ-*superstring for* (*H*, *T*, *w*) *is a string* **v** ∈ A* *such that for every* **h** ∈ *H, the sum of the weights of the target strings that are common substrings of both* **h** *and* **v** *is at least* λ.

More formally, denoting by *CS*(**s**, **t**) the set of all common substrings of two strings **s** and **t**, a weighted λ-superstring for (*H*, *T*, *w*) is a string **v** ∈ A* such that

$$\begin{array}{c} \sum_{t\in CS(h,v)\cap T}w\left(t\right)\geq \lambda \text{for}\;\text{all}\;\text{h}\in H\;. \end{array}$$

Clearly, if *w*(**t**) = 1 for all **t** ∈ *T*, then a string **v** is a weighted λ-superstring for (*H*, *T*, *w*) if and only if **v** is a λ-superstring for (*H*, *T*).

The corresponding optimization problem (Box 1) is the following:

**Box 1**

Shortest Weighted λ-Superstring

**Instance**: A finite set of *H* ⊆ A* of *host* strings, a finite set of *T* ⊆ A* of *target* strings, a weight function w:T→R, a *covering requirement* λ ∈ *R*.

**Task**: Find a weighted λ-superstring for (*H*, *T*, *w*) of minimum length.

The restriction of the Shortest Weighted λ-Superstring problem to instances such that *w*(**t**) = 1 for all **t** ∈ *T* is equivalent to the Shortest λ-Superstring problem defined in [10].

**Definition 2** *Let* C *be a collection of finitely many finite sets of strings over A, let* $T=\cup_{X\in C}X$, *let* w:T→R, *and let* λ∈R. *A* weighted λ-cover superstring *for* (C,w) *is a string* **v** ∈ A* *such that for every* X∈C, *the sum of the weights w*(**t**) *of the strings* **t** ∈ *X that are substrings of* **v** *is at least* λ. *Formally, for every* X∈C, *we have* ∑_**t**∈*X*,**t**⊆**v**_ *w*(**t**) ≥ λ.

Clearly, the case of unit weights corresponds to the notion of a λ-cover superstring. The corresponding optimization problem (Box 2) is the following:

**Box 2**

Shortest Weighted λ-Cover Superstring

**Instance**: A collection C of finitely many finite sets of finite strings over alphabet *A*, a weight function $w:\cup_{X\in C}X\to R$, a *covering requirement* λ∈R.

**Task**: Find a weighted λ-cover superstring for (C,w) of minimum length.

The restriction of the Shortest Weighted λ-Cover Superstring problem to instances such that *w*(**t**) = 1 for all $t\in \cup_{X\in C}X$ is equivalent to the Shortest λ-Cover Superstring problem defined in [10]. In that paper, it was proved that the Shortest λ-Superstring problem is polynomially equivalent to the Shortest λ-Cover Superstring problem. This equivalence extends straightforwardly to the weighted versions of the problems. Moreover, since the weighted versions of the problem generalize the unweighted ones, hardness results from [10] immediately carry over to the weighted ones. In particular:

**Theorem 3** *1. For every ϵ* > 0, *there is no polynomial time algorithm approximating the* Shortest Weighted λ-Superstring *problem within a factor of* (1 − *ϵ*)ln |*H*|, *unless* P = NP, *even for the case of the binary alphabet A* = {0, 1}, *a constant weight function w* ≡ 1, *and* λ = 1.

*2. For every ϵ* > 0, *there is no polynomial time algorithm approximating the* Shortest Weighted λ-Cover Sperstring *problem within a factor of* (1-ϵ)ln|C| *unless* P = NP, *even for the case of the binary alphabet, a constant weight function w* ≡ 1, *and* λ = 1.

The corresponding hardness results from [10] are stated with a multiplicative constant of *c* > 0.2267 instead of 1 − *ϵ*. However, exactly the same approach as the one used to prove Theorem 3.9 and Corollary 3.10 in [10] can be used to derive Theorem 3; one only needs to use the more recent, stronger inapproximability result on the set cover problem due to Dinur and Steurer [14] instead of the one due to Alon et al. [15].

Theorem 3 suggests that most likely the two problems cannot be solved optimally or approximately by efficient algorithms, and motivate the development of exact exponential time algorithms and of suboptimal heuristic approaches. This is what we do in the next two subsections.

### Graph theoretic and integer programming formulations of the shortest weighted λ-cover superstring problem

In this section, we extend the graph theoretic and integer programming (IP) formulations of the Shortest λ-Cover Superstring problem from [10] to the weighted case. (For background on integer programming, see, e.g., [16]). Following [10], we model the problem as a generalization of the *generalized Traveling Salesman Problem*. In this problem, the set of vertices of a given complete directed edge-weighted graph is divided into clusters and the objective is to find a minimum-cost tour passing through at least one node from each cluster.

The graph theoretic model for the Shortest λ-Cover Superstring problem from [10] is based on a derived complete edge-weighted directed graph *G* with vertex set $T=\cup_{X\in C}X$ plus one special vertex. Roughly speaking, the main idea is the following. Given a λ-cover superstring **v** for C, one can identify a set of substrings of **v** that are pairwise incomparable with respect to the substring relation and contain, as substrings, at least λ strings from each cluster X∈C. Sorting these strings in order of their first appearance in **v** yields a directed path in *G* that can be extended to a directed cycle in *G* through the special vertex. By construction, the vertices of this cycle “cover” (in the sense of substring relation, when viewed as strings) at least λ vertices from each cluster X∈C. The weights of the edges are defined so that the length of the resulting cycle does not exceed the length of **v**. And conversely, every directed cycle in *G* through the special vertex satisfying the above covering property and such that no two strings corresponding to (non-special) vertices of the cycle are comparable with respect to the substring relation can be transformed into a λ-cover superstring **v**, by taking the overlapping sum of the strings corresponding to the non-special vertices of the cycle. The weights of the edges are defined so that the length of the cycle equals the length of the obtained superstring.

We now formalize these notions and explain the extension to the weighted case. Consider an instance (C,w,λ) of the Shortest Weighted λ-Cover Superstring problem, and let $T=\cup_{X\in C}X$. Following [10], we construct a complete directed edge-weighted graph *G* = (*V*, *E*, *c*), called the *distance graph*. To distinguish the edge weights from the weights from the input weight function *w*, the weights on edges will also be referred to as *costs* and will be specified with a function $c:E\to Z_{+}$. The construction is the same as in [10]:

- *V* = *T* ∪ {s*}.
- For every two distinct vertices *s*, *t* ∈ *T*, add the arc (*s*, *t*) to *E* and assign to it the cost *c*(*s*, *t*) = *ℓ*(*s*) − *ov*(*s*, *t*). Clearly, the costs are well defined and non-negative.
- For every vertex *s* ∈ *T*, add the arc (*s*, s*) to *E* and assign to it cost *c*(*s*, s*) = *ℓ*(*s*).
- For every vertex *s* ∈ *T*, add the arc (s*, *s*) to *E* and assign to it zero cost, *c*(s*, *s*) = 0.

We emphasize that in what follows, we identify the vertices of *G* other than s* with the corresponding strings from *T*. In particular, for *i*, *j* ∈ *V*(*G*) \ {s*}, notation *i* ⊆ *j* means that *i* is a substring of *j* and *i* ⊂ *j* that *i* is a proper substring of *j*. One more definition is needed to express the problem as a graph problem. A subgraph *H* of *G* is said to *cover* a string **s** ∈ *T* if there exists a vertex **t** ∈ *V*(*H*) ∩ *T* such that **s** ⊆ **t**. For X∈C, we will denote the set of all strings in *X* covered by *H* by *X*_*H*_. The *cost* of a directed cycle *C* in *G* is defined as ∑_*e*∈*E*(*C*)_ *c*(*e*).

**Definition 4** *A directed cycle C in the distance graph G is said to be w*-feasible *if it satisfies the following conditions*:

1. s* ∈ *V*(*C*).
2. *For every two distinct vertices* **s**, **t** *from V*(*C*) ∩ *T*, **s** *is not a substring of* **t**.
3. *For every* X∈C, *we have* $\sum_{t\in X_{C}}w\left(t\right)\geq \lambda$.

**Proposition 5** *Let* (C,w,λ) *be an instance to the* Shortest Weighted λ-Cover Superstring *problem, and let G be its derived distance graph. Then, there exists a weighted* λ-*cover superstring for* (C,w) *of length at most ℓ if and only if G contains a w-feasible directed cycle C of cost at most ℓ*.

We give a proof of Proposition 5 in S1 Appendix.

Proposition 5 leads to the following IP formulation for the Shortest Weighted λ-Cover Superstring problem. The program has three types of binary variables: *x*_*ij*_, where (*i*, *j*) ranges over all ordered pairs of distinct elements of *V*, *y*_*i*_, where *i* ranges over all elements of *V*, and *z*_*i*_, where *i* ranges over all elements of *T*. Recall that $c:E\to R_{+}$ is the cost function on the edges of the distance graph *G*.

$$\begin{array}{ccc} \min & \;\sum_{i,j}c\left(i,j\right)x_{ij} & \\ s.t. & \;y_{s^{*}}=1 & \\ & \;\sum_{i\in V\;:\;i\neq j}x_{ij}=y_{j} & \;\forall j\in V\\ & \;\sum_{j\in V\;:\;j\neq i}x_{ij}=y_{i} & \;\forall i\in V\\ & \;\sum_{i\in X}w\left(i\right)z_{i}\geq \lambda & \;\forall X\in C\\ & \;\sum_{i\subseteq j}y_{j}\geq z_{i} & \;\forall i\in T\\ & \;y_{i}+y_{j}\leq 1 & \;\forall i,j\in T\text{such}\;\text{that}\;i\subset j\\ & \;0\leq x_{ij}\leq 1, & \;x_{ij}\;\text{integer}\\ & \;0\leq y_{i}\leq 1, & \;y_{i}\;\text{integer}\\ & \;0\leq z_{i}\leq 1, & \;z_{i}\;\text{integer} \end{array}$$

The feasible solutions of the IP described above are in correspondence with subgraphs *H* of *G* containing s* that consist of one or more *subtours* (vertex-disjoint directed cycles) in which the vertices other than s* correspond to a set of strings that are pairwise incomparable with respect to the substring relation and such that the covering requirement

$$\begin{array}{c} \sum_{t\in X_{H}}w\left(t\right)\geq \lambda \;. \end{array}$$

is satisfied.

To be able to apply Proposition 5, we are only interested in solutions that consist of a single directed cycle. As discussed in [10], this can be achieved in several ways (see, e.g., [17]), for instance using the Miller-Tucker-Zemlin (MTZ) formulation [18], the subtour formulations, or with a combined approach resulting in a cutting-plane algorithm.

**Fig 1:**
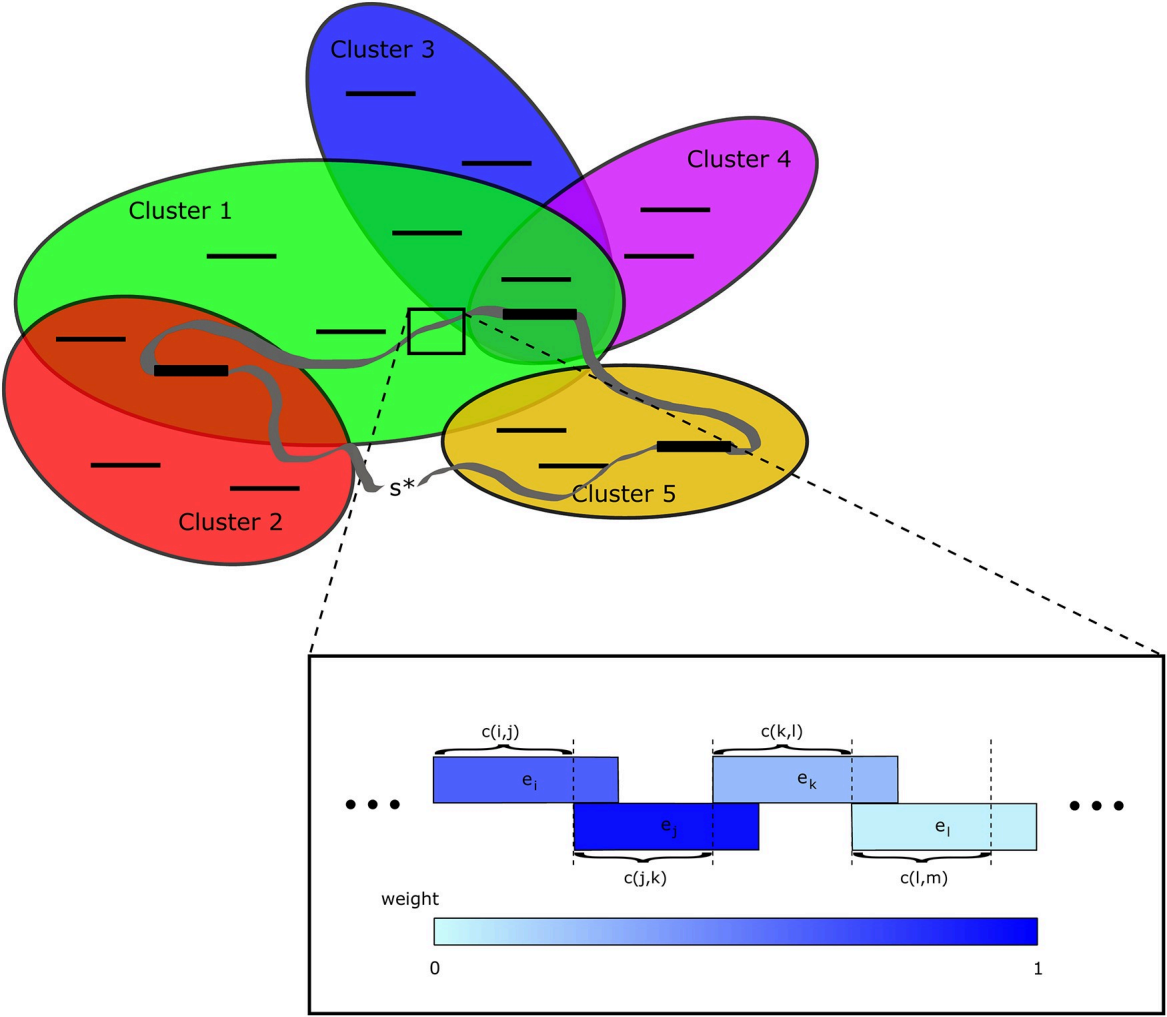
Graphical interpretation of the connection of the combinatorial optimization problem to the graph problem. The clusters associated to the host strings are shown in ovals with the corresponding target strings inside them. Each target string has an associated weight, which is shown in this example using a color code from light blue to strong blue, with extreme values corresponding to 0 and 1, respectively. The λ-superstring is represented with a closed ribbon which travels among the clusters. It is closed because one of the strings forming it corresponds to the artificial vertex s*, which is not a host string, but can be viewed as an empty string gluing the extremes of the λ-superstring. The condition that for each one the clusters, the sum of the weights of the target strings that are both in the λ-superstring and in the cluster is at least λ is imposed in the feasible solutions. The length of the λ-superstring is minimized, and this length can be obtained by summing up the *c*(*i*, *j*) values of the strings forming the λ-superstring. The *c*(*i*, *j*) values are shown in the figure as the length of the part of the vertex labelled by *i* not overlapping with the next vertex in the λ-superstring, which is labelled by *j*.

In Fig 1, we represent an ilustrative sketch linking the combinatorial optimization problem to the graph problem.

### A genetic algorithm

In this section we will present a genetic algorithm well suited to find solutions to a problem with potential applications to vaccine design posed, for unweighted λ-superstrings, in the concluding section of [10]. The problem is the following: Given a set of host strings of approximately similar lengths corresponding to the same protein with different mutations in a set of patients, find a λ-superstring of about one-gene length with λ as big as possible when the set *T* of target strings is formed by all the substrings of a given length *ℓ* of the set of host strings, while keeping, as much as possible, the relative order of the elements in *T*. In other words, the goal is to design a synthetic protein enriched in the sense that it covers many epitopes in each host string. In our more general setting of weighted λ-superstrings we require these epitopes to be very immunogenic. As the second objective of our multi-objective optimization program, we have chosen to optimize the amino acid resemblance with the virus peptides. By using the alignment as target to be optimized, we will be able to choose candidates that have a structure similar to those which already interacted with HIV patients, and therefore will likely be recognized by the immune system.

We opt for a genetic algorithm in this case because the high number of target strings makes the use of integer programming impractical; employing heuristic methods of optimization is thus a good alternative. We do sacrifice on optimality; nevertheless, suboptimal solutions can be satisfactory in practice.

We are faced with a multi-objective optimization problem. To solve such problems, multi-objective functions *f*: *P* → *R*^*n*^ are considered, where *P* is the set of feasible solutions, that assign to each element *x* ∈ *P* an *n*-tuple (*f*_1_(*x*), …, *f*_*n*_(*x*)) with real entries, each of which indicates a partial objective function. Without loss of generality, we can assume that we want to *maximize* each partial objective function, because minimizing *f*_*i*_(*x*) is equivalent to maximizing the opposite function −*f*_*i*_(*x*). Obviously, it is not possible in general to get a solution *x* ∈ *P* in which all partial objective functions *f*_*i*_ attain maximum value. Instead, optimality of a solution is established in terms of *Pareto domination*: given two feasible solutions *x*, *y* ∈ *P*, we say that *x* = (*x*_1_, …, *x*_*n*_) is dominated by *y* = (*y*_1_, …, *y*_*n*_) if *x*_*i*_ ≤ *y*_*i*_ for every *i* and *x*_*j*_ < *y*_*j*_ for some *j*. The *Pareto front* is formed by the elements in *P* which are not dominated by any element of *P*.

Very often evolutionary algorithms are used to evolve an initial population *P*_0_ ⊆ *P* to obtain a sequence *P*_*i*_ of populations which get closer to the Pareto front, and it is desirable to obtain wide-spread sets of solutions. In particular, several genetic algorithm approaches have been proposed for these kinds of problems. One of the most reliable and quick ones among them is NSGA-II [11], and we have used it for our optimization problem. For definitions and results on genetic algorithms we refer the reader to [19].

We outline here the structure of the NSGA-II algorithm. We refer to [11] for details.

Given a set *P*′ ⊆ *P* of feasible solutions, two key values are assigned to each *x* ∈ *P*′: the non-domination rank *x*_rank_ and the crowding distance *x*_distance_. The process of assignment of non-domination ranks is as follows. The non-dominated elements, that is, the elements in the Pareto front of *P*′ are assigned rank 1, and they form the set *F*_1_. If we take *P*′ − *F*_1_, the non-dominated elements in this set are assigned rank 2, and they form the set *F*_2_, and so on. This ordering is done using the fast non-dominated sorting described in [11]. The crowded distance *x*_distance_ is calculated by taking the average distance of two points on either side of *x* along each of the *n* objectives. This leads to a strict partial order on *P*′ defined by

$$\begin{array}{c} x≺y\;\text{if}\;x_{\text{rank}}<y_{\text{rank}}\;\text{or}\;\text{if}\;x_{\text{rank}}=y_{\text{rank}}\;\text{and}\;x_{\text{distance}}>y_{\text{distance}}. \end{array}$$

The general process in NSGA-II is as follows:

First, given a parameter *m*, a random population *P*_0_ of size *m* is constructed, and it is sorted according to the relation ≺ defined above. Then, a binary tournament selection is done considering the relation ≺. In the tournament selection it is theoretically possible, although it is unlikely, that two different elements are not comparable with respect to the relation, because they have the same rank and the same crowded distance. In this case, one of them is chosen uniformly at random. After the tournament selection is completed, mutation and crossing is done on the selected elements, to create an offspring population *Q*_0_ of size *m*. Now a combined population *R*_0_ = *P*_0_ ∪ *Q*_0_ is formed, and the elements in *R*_0_ are sorted according to their domination level. Then, a new population *P*_1_ is formed by collecting the elements in *R*_0_ in ascending order of ranks, that is, we take the elements in the set *F*_1_ formed by the elements of rank 1, then the elements in *F*_2_, and so on, until all the elements of a certain set *F*_*i*−1_ have been allocated but there is no place to allocate all the elements of *F*_*i*_, that is, until |*F*_1_∪ ⋯ ∪*F*_*i*−1_|≤*m* but |*F*_1_∪ ⋯ ∪*F*_*i*_| > *m*. Then, we rank the elements in *F*_*i*_ according to its crowding distance and we select elements in non-increasing order of crowding distance until we have *m* elements in *P*_1_. Now, given a parameter *niter*, the process is iterated *niter* times to obtain a population *P*_*i*+1_ from a population *P*_*i*_ for any *i* in the same way that we obtained *P*_1_ from *P*_0_.

Next we will describe how we use NSGA-II for our particular problem.

We want to find a weighted λ-superstring for a set *H* = {**h**_1_, …, **h**_*spop*_} of host strings, a set *T* of target strings formed by all the subsequences of a given length *ℓ* of the strings of *H*, and a weight mapping *w* assigning real values to elements of *T*. The chromosomes in the genetic algorithm will be sequences of target strings. The phenotype of a chromosome *u* will be the overlapping sum *o*(*u*) of the target strings which constitute it (according to the sequence in which they appear in *u*). The fitness function that we consider for each chromosome *u* in the population is taken to be *f*(*u*) = (λ(*u*), *al*(*u*)), where:

- λ(*u*) is an estimate of the maximum value for which *o*(*u*) is a weighted λ(*u*)-superstring for (*H*, *T*), defined by
  $$\begin{array}{c} \lambda \left(u\right)=\min\{\sum_{t\in u,t\;\text{substring}\;\text{of}\;{\text{h}}_{i}}w\left(t\right):i=1,\ldots ,spop\} \end{array}$$
  (it is an estimate because the true value of the maximum λ could, in principle, be different from λ(*u*) if there are elements of *T* covered by *o*(*u*) which are not in *u*), and
- *al*(*u*) is the average value of the scorings for the pairwise global alignments of *o*(*u*) and each of the strings **h**_*i*_.

The specific scoring scheme may depend on the application; in the Results section we specify it for our particular biological application. (For background on string alignment, see [20]).

We have used a modified version of NSGA-II in which we take the *Q*_*i*_ sets of a cardinality *m* greater than *spop*, so that |*R*_*i*_| > 2|*P*_*i*_| for every *i*. Also, instead of taking the initial population *P*_0_ randomly, we have taken it to be formed by the sequences of target strings corresponding to the set {**h**_1_, …, **h**_*spop*_} of host strings, in the order of appearance in each host string.

For the crossing of two chromosomes $\left(u_{1},\ldots ,u_{\ell_{1}}\right)$ and $\left(v_{1},\ldots ,v_{\ell_{2}}\right)$, we have used a one-point crossing in which we select randomly a crossing point *c* between 1 and min{*ℓ*_1_, *ℓ*_2_} − 1 and take $\left(u_{1},\ldots ,u_{c},v_{c+1},\ldots ,v_{\ell_{2}}\right)$ as the first child and $\left(v_{1},\ldots ,v_{c},u_{c+1},\ldots ,u_{\ell_{1}}\right)$ as the second child.

Once the crossing has been done, we have assigned a probability of mutation *prmut* in each gene of each child chromosome. A mutation in the *i*-th position of a chromosome $u=(u_{1},\ldots ,u_{\ell_{1}})$ is done by selecting first a random integer *j* obtained by rounding a real number sampled according to the normal distribution with mean *i* and standard deviation defined by a parameter *sd*, choosing then uniformly at random a sequence $\left(v_{1},\ldots ,v_{\ell_{2}}\right)$ associated to a host string from the initial population and substituting *u*_*i*_ with *v*_*j*_ in the chromosome *u* if 1 ≤ *j* ≤ *ℓ*_2_; in any other case, the mutation is not done. The idea of this mutation that we have just described is to substitute the *u*_*i*_ with an element ‘not far from the *i*-th position’, in the sense that it is close to an element in the *i*-th position in a chromosome of the initial population formed by the host strings.

## Results

In this section, an application of the IP-based algorithm and of the genetic algorithm is given to find weighted λ-superstrings for a set of 169 host strings whose GenBank [21] access numbers appear in S1 Table, corresponding to the Nef protein, and two sets of target strings (epitopes) chosen in a way that will be made clear soon. The 169 sequences were from HIV-1 subtype B independently infected individuals, and this specific set was first considered by Nickle et al. in [22], and later by our group in [10]. Thus, we used this same set in order to be able to compare the method here proposed, to our previous work [10]. This comparison can be found at the end of this section.

### Applying the integer programming formulation

We begin with the IP-based algorithm described in the Materials and methods section. We consider the set of epitopes shown in S2 Table.

The weights corresponding to the immunogenicities of epitopes were experimentally obtained from the data appearing in the Immune Epitope Database and Analysis Resource (IEDB) [23]. We selected the epitopes for the Nef protein satisfying simultaneously the following three conditions:

1. they are covered by at least one of the 169 host strings analyzed;
2. they appear in the HIV Molecular Immunology Database [24];
3. they appear in IEDB with a positive value of *p* + *n*, where *p* and *n* are the number of positive and negative results, respectively, in the MHC Ligand Assays section.

We took the ratio *p*/(*p* + *n*) as the weighting of the epitopes. Note that a non-linear rescaling of the weights (i.e., normalizing them) would change the optimization problem. However, we consider that to justify a rescaling we would require empirical evidence pointing that the candidates give better results, and that is out of the scope of this work. The main reason for considering this weighting is that the empirical response of an epitope can only be verified through assays, so we estimated it numerically by the aforementioned ratio. Moreover, we used the MHC Ligand Assays, because there are several works stating that there exists a correlation between the generated immune response and MHC complex stability [25] or MHC affinity [26], and it has been used to predict T Cell epitopes [27]. The values are also shown in S2 Table.

The solutions found with the IP-based algorithm and the values of the corresponding parameters are shown in Tables 1 and 2, which we now explain.

**Table 1:** Optimal solutions of minimum length for a given value of λ.

λ = 1.0
Optimal λ superstring: TQGYFPDWQNYVPLRPMTYPLTFGWCF
Optimal λ superstring length: 27
Covering value of the solution: 1
λ = 1.5
Optimal λ superstring: LTFGWCFKLVFPVRPQVPLRPMTYKAAVDLSHFLK
Optimal λ superstring length: 35
Covering value of the solution: 1.51
λ = 1.9
Optimal λ superstring: KAAVDLSHFLTFGWCFKLVFPVRPQVPLRPMTYTQGYFPDWQNY
Optimal λ superstring length: 44
Covering value of the solution: 1.94
λ = 2
Optimal λ superstring: KAAVDLSHFLKLTFGWCFKLVFPVRPQVPLRPMTYTQGYFPDWQNY
Optimal λ superstring length: 46
Covering value of the solution: 2
λ = 2.5
Optimal λ superstring: TQGYFPDWQNYPLTFGWCFKLVFPVRPQVPLRPMTYKAAVDLSHFLK
Optimal λ superstring length: 47
Covering value of the solution: 2.51
λ = 2.6
Optimal λ superstring: FPVRPQVPLRPMTYKAAVDLSHFLKEKGGLTQGYFPDWQNYTPGPGVRYPLTFGWCFKLV
Optimal λ superstring length: 60
Covering value of the solution: 2.68
λ = 2.9
Optimal λ superstring: TPGPGVRYPLFPVRPQVPLRPMTYKAAVDLSHFLKTPGPGIRYPLTFGWCFKLVTQGYFPDWQNY
Optimal λ superstring length: 65
Covering value of the solution: 2.94
λ = 3.2
Optimal λ superstring: TPGPGIRYPLTPGPGVRYPLTFGWCFKLVPEKEVLVWKFDSRLAFHHQEILDLWVYFPVRPQVPLRPMTYKAAVDLSHFLKEKGGLEGLTQGYFPDWQNY
Optimal λ superstring length: 100
Covering value of the solution: 3.25

**Table 2:** Optimal solutions with maximum λ for a given upper bound on the length of the string.

Upper bound on string length = 10
Optimal value of λ = 0.0
Optimal λ superstring: AVDLSHFL
Optimal λ superstring length: 8
Upper bound on string length = 20
Optimal value of λ = 0.0
Optimal λ superstring: AVDLSHFL
Optimal λ superstring length: 8
Upper bound on string length = 30
Optimal value of λ = 1.0
Optimal λ superstring: TQGYFPDWQNYPLTFGWCFQVPLRPMTYK
Optimal λ superstring length: 29
Upper bound on string length = 40
Optimal value of λ = 1.51
Optimal λ superstring: LTFGWCFKLVFPVRPQVPLRPMTYKAAVDLSHFLKEKGGL
Optimal λ superstring length: 40
Upper bound on string length = 50
Optimal value of λ = 2.51
Optimal λ superstring: TQGYFPDWQNYPLTFGWCFKLVFPVRPQVPLRPMTYKAAVDLSHFLK
Optimal λ superstring length: 47
Upper bound on string length = 60
Optimal value of λ = 2.68
Optimal λ superstring: TQGYFPDWQNYTPGPGVRYPLTFGWCFKLVFPVRPQVPLRPMTYKAAVDLSHFLKEKGGL
Optimal λ superstring length: 60
Upper bound on string length = 70
Optimal value of λ = 2.94
Optimal λ superstring: TPGPGIRYPLTQGYFPDWQNYTPGPGVRYPLTFGWCFKLVFPVRPQVPLRPMTYKAAVDLSHFLKEKGGL
Optimal λ superstring length: 70
Upper bound on string length = 80
Optimal value of λ = 2.94
Optimal λ superstring: TPGPGIRYPLTQGYFPDWQNYTPGPGVRYPLTFGWCFKLVFPVRPQVPLRPMTYKAAVDLSHFLKEKGGL
Optimal λ superstring length: 70
Upper bound on string length = 90
Optimal value of λ = 2.94
Optimal λ superstring: TPGPGIRYPLTQGYFPDWQNYTPGPGVRYPLTFGWCFKLVFPVRPQVPLRPMTYKAAVDLSHFLKEKGGL
Optimal λ superstring length: 70
Upper bound on string length = 100
Optimal value of λ = 3.25
Optimal λ superstring: QEILDLWVYTQGYFPDWQNYTPGPGIRYPLPEKEVLVWKFDSRLAFHHTPGPGVRYPLTFGWCFKLVFPVRPQVPLRPMTYKAAVDLSHFLKEKGGLEGL
Optimal λ superstring length: 100

In the analysis whose results are shown in Table 1, the value of λ was varied from 1.0 up to 3.3 in increments of 0.1, and for each value of λ, the total length of the λ-superstring was minimized. Solutions were obtained by implementing the integer program descrived in Materials and Methods (extended with the MTZ formulation) in Java [28] and solving it to optimality using IBM ILOG CPLEX Optimization Studio [29]. The integer program corresponding to the case λ = 3.3 turned out to be infeasible; all the others were feasible. In the table we also show the *covering value* of the obtained solution, that is, the value of $\min_{X\in C}\sum_{i\in X}w\left(i\right)z_{i}$ (using notation from the Materials and methods section). Only the results not dominated by others are shown, in the sense that in cases when for different values of λ the same optimal solution strings were found, only the highest value of λ is shown.

Table 2 shows the results of a “dual” analysis in which we were maximizing the value of λ subject to imposing an upper bound on the length of a λ-superstring for the given sets of host and target strings. The results were obtained by solving a straightforward modification of integer program (and its extension with the MTZ formulation), again using Java and CPLEX. The modification of the IP consists in treating λ as a variable, replacing the objective function ∑_*i*,*j*_ *c*(*i*, *j*)*x*_*ij*_ with λ and min with max, and adding the constraint ∑_*i*,*j*_ *c*(*i*, *j*)*x*_*ij*_ ≤ *ℓ*, where *ℓ* is a given upper bound on the string length. Clearly, since we are maximizing λ, in any optimal solution the value of λ will be equal to the covering value, that is, $\lambda =\min_{X\in C}\sum_{i\in X}w\left(i\right)z_{i}$ (again, using notation from the Materials and methods section).

The upper bound *ℓ* on the length of the λ-superstring was varied from 10 to 200 in increments of 10. Increasing the upper bound on the string length from 100 to anywhere up to 200 did not result in any increase in the covering value λ. We therefore only display in Table 2 the results for the values of the upper bounds up to 100. Since in this second model the length of the obtained solution was only constrained by an upper bound and not taken into account in the objective function, it should not be surprising that the corresponding solutions found for upper bounds between 100 and 200 were of different lengths, despite the fact of being equally good in terms of their covering values. A similar phenomenon occurred also for values of the upper bound *ℓ* displayed in the table: the optimal covering values of the solutions corresponding to the upper bounds in each of the ranges 10–20 and 70–90 were the same.

We are interested in high covering values while keeping the length of the λ-superstring small. It is therefore interesting to analyze which of the solutions found by the above analysis have the best (that is, highest) ratio between the covering value and the length. In this respect, the best solution found by the above analysis is the λ-superstring of length 47 achieving a covering value of 2.51 (see Table 1). The same covering value is also achieved by the string of length 47 shown in Table 2. Only slightly worse ratios were achieved by the solutions from the above tables corresponing to the following (length, covering value) pairs: (44, 1.94), (60, 2.68), (65, 2.94) (all from Table 1).

Another aspect of such analysis that might be potentially interesting for vaccine design applications would be to identify the maximum possible covering value that can be achieved for a given set of host and target strings (without any restriction on the length of the λ-superstring), and then find a shortest substring realizing this covering value. In the instance analyzed above, this maximum covering value is equal to 3.25, and the shortest length of aλ-superstring achieving this covering value is 100.

### Applying the multiobjective genetic algorithm

We used the NSGA-II multiobjective genetic algorithm described in the Materials and methods section for the same set of 169 host strings used in the previous subsection whose GenBank IDs appear in S1 Table. The set of target strings was taken to be the set of all 9-mers present in the host strings. Unlike in the previous subsection, immunogenicities were not obtained experimentally, because of the technical difficulty and the high cost of estimating empirically the immunogenicity of a large number of sequences. In this case, the immunogenicity associated to each of the target strings (that is, the value of the weight function *w*(**t**)) was computationally assessed.

Several algorithms to estimate numerically the immunogenicity of epitopes have been proposed in the literature, see, for instance, [30–39]. We selected in our analysis the algorithm proposed in [34], where a tool was also given in the “T-cell” epitopes-Immunogenicity Prediction” of the “IEDB Analysis Resource” [40].

We ran the genetic algorithm by using the program Mathematica [41] with the following set of parameters:

$$\begin{array}{c} niter=500,spop=169,prmut=0.01,m=1352\;\text{and}\;sd=1. \end{array}$$

We used the Mathematica command NeedlemanWunschSimilarity, which gives the number of one-element matches in the alignment, for calculating the scorings of the global alignments that are averaged to obtain the values of *al*(*u*) described in the Materials and methods section.

**Fig 2:**
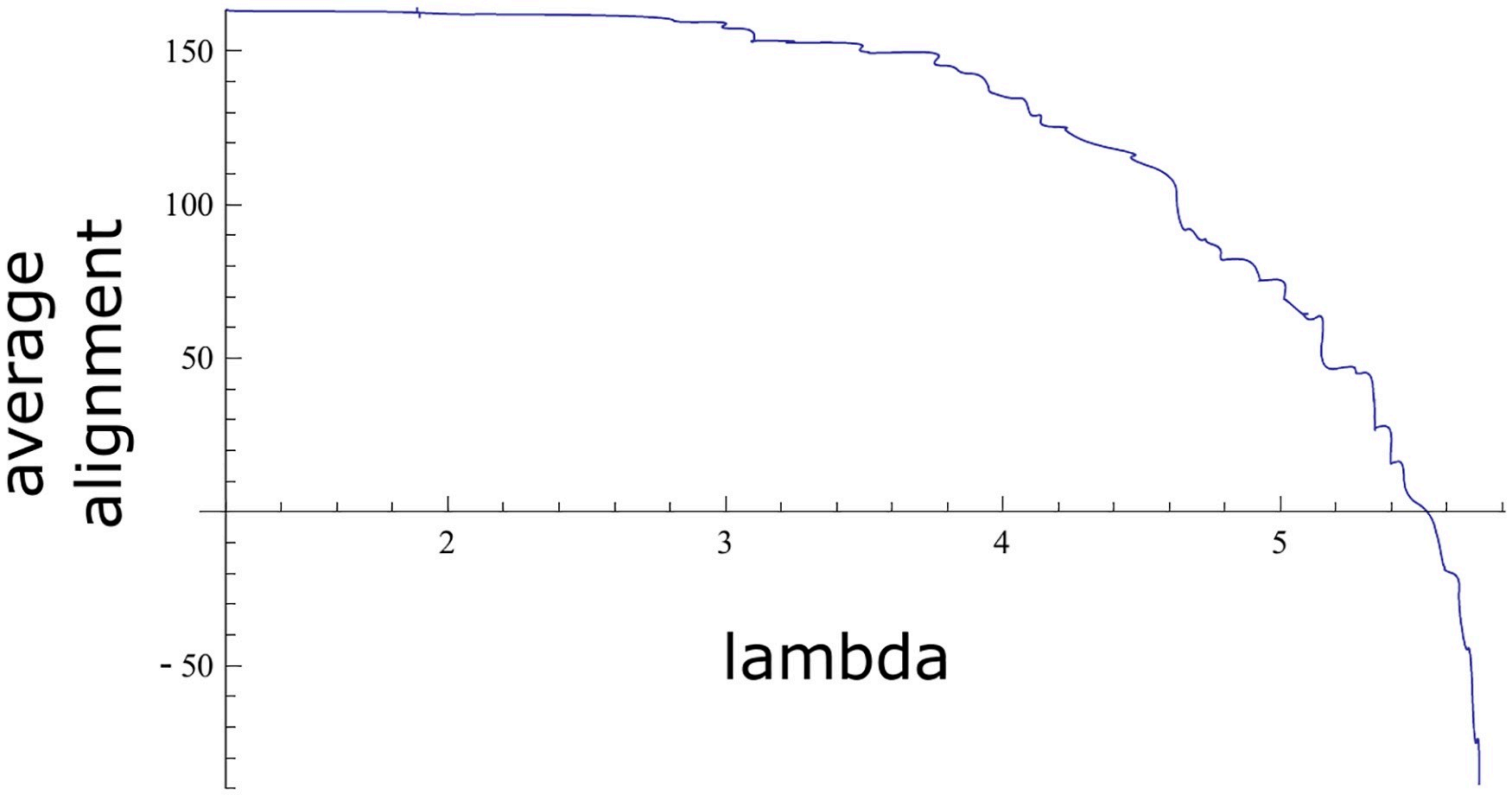
Estimation of the Pareto front in the genetic algorithm. The line represents the non-dominated solutions found with the genetic algorithm. The X axis indicates the λ value, while the Y axis indicates the alignment score.

We run 20 times the NSGA-II algorithm and collected the non-dominated solutions obtained in each of the runs. We eliminated the dominated solutions to obtain a final estimation of the Pareto front. The values are shown in Fig 2 and in Table 3. The resultant estimation of the Pareto front gave a set of non-dominated sequences with a maximum λ of 5.71 and a minimum value of 1.2 (average ± SD of 4.32±1.14). The alignments ranged between -88.47 and 163.33 (average± SD of 87.73±67.43). The distributions of λ and the alignment values are represented in S1 Fig panel (a) and (b), respectively.

**Table 3:** Numerical values for the estimation of the Pareto front in the genetic algorithm.

(1.2017,163.33)	(3.7513,149.3)	(4.6507,92.16)	(5.3242,44.29)
(1.293,162.95)	(3.7547,145.87)	(4.6771,91.94)	(5.3374,36.46)
(1.7287,162.79)	(3.8153,144.98)	(4.7089,88.77)	(5.3391,36.19)
(1.8909,162.49)	(3.855,142.96)	(4.7308,88.71)	(5.3413,27.89)
(1.8977,162.44)	(3.9156,142.07)	(4.7392,87.72)	(5.3492,27.59)
(2.0255,161.96)	(3.9461,138.79)	(4.7815,85.76)	(5.3959,26.6)
(2.1794,161.93)	(3.9526,136.87)	(4.787,82.31)	(5.3974,17.13)
(2.5507,161.58)	(3.9797,135.85)	(4.8195,82.26)	(5.4096,16.14)
(2.7806,160.63)	(4.0195,134.82)	(4.8925,81.32)	(5.4404,15.24)
(2.8411,159.48)	(4.0502,134.62)	(4.9253,76.17)	(5.4582,6.15)
(2.9918,159.25)	(4.08,133.92)	(4.9355,75.31)	(5.5322,-0.18)
(2.9923,157.56)	(4.0977,129.8)	(5.0094,75.04)	(5.57,-9.33)
(3.0863,156.8)	(4.1161,128.93)	(5.0147,69.88)	(5.585,-16.12)
(3.1024,153.34)	(4.1377,128.84)	(5.0249,68.67)	(5.5929,-18.14)
(3.1083,153.31)	(4.1458,125.92)	(5.078,64.63)	(5.5988,-19.13)
(3.2322,153.22)	(4.2259,125.13)	(5.0834,64.36)	(5.6403,-21.67)
(3.2357,152.79)	(4.2286,124.14)	(5.1092,62.62)	(5.6454,-30.74)
(3.2449,152.69)	(4.3204,120)	(5.1528,62.41)	(5.6671,-43.62)
(3.4688,152.4)	(4.4694,116.65)	(5.1562,48.33)	(5.6859,-47.61)
(3.4855,150.26)	(4.47,114.67)	(5.2502,47.24)	(5.6998,-72.72)
(3.5152,149.62)	(4.602,108.9)	(5.2719,46.02)	(5.7141,-74.6)
(3.546,149.37)	(4.6296,98.75)	(5.2798,45.08)	(5.717,-88.47)

We selected and analyzed in the estimation of the Pareto front the solution with scoring value 161.93 and λ value 2.1794. We have chosen this sequence due to several reasons. First, theλ and the scoring are greater than the ones of all the members in the initial population of 169 strings, for which the mean of the λ values was -1.70395, the maximum λ value was 1.59422, the mean of the scores was 143.34 and the maximum score was 157.66. Second, there is a remarkable level of maintenance of the highly conserved regions of the protein for this solution. Nonetheless, other solutions in the estimation of the Pareto front with greater values of λ and lower scorings could, of course, be useful in practice.

The sequence of amino acids of the selected solution is

MGGKWSKRSGVGWPTVRERMRRAEPAADGVGAV
SRDLEKHGAITSSNTAATNADCAWLEAQEEEEVGF
PVRPQVPLRPMTYKAAVDLSHFLKEKGGLEGLIYS
QKRQDILDLWIYHTQGYFPDWQNYTPGPGIRYPLT
FGWCFKLVPVEPEKVEEANEGENNSLLHPMSLHG
*MEDPEKEVLEWKFDSRLAFHHMARELHPEYYKDC*.

Since the main goal in this section is to study the structure and functionality of a protein modelled by a sequence with given fixed values of λ and of the average score, we performed several bioinformatics analyses to the string showed in the previous paragraph.

The average value of the lengths of the 169 sequences whose GenBank IDs appear in S1 Table is 207.11, and the length of our sequence is 206, which is very close to that average. In fact, 206 is the length established for Nef in [42], where the distribution of the amino acids of 1643 Nef sequences was analyzed. This does not imply, of course, that the protein has a well-defined length (there are deletions and insertions in certain positions for some of the sequences) and there is not the same amino acid residue for each position in all sequences. Given the high variability of the protein, it is more appropriate to see the protein as a non-deterministic distribution of residues conserving to some extent the secondary and tertiary structures and the functionality.

In order to study to what extent our solution captures the well conserved regions of the protein, we considered the sequences of residues conserved at 90% and their starting positions searching in the table of O’Neill et al. [42, Fig 1]. The sequences and positions are shown in S3 Table.

In our solution, all the sequences appear at the same positions as in the table, so all the oligopeptides conserved at 90% are kept.

In order to analyze the structure of the candidate sequence, we have used the bioinformatics tool I-TASSER [43], [44], which is an open source software implemented by Zhang Lab—University of Michigan.

Among the available software, we chose I-TASSER because it has ranked several years as the top method in Critical Assessment of protein Structure Prediction (CASP) experiment, a worldwide test which every two years evaluates the protein structure prediction methods proposed by research groups. More precisely, I-TASSER ranked n°1 in CASP7 (2006), CASP8 (2008), CASP9 (2010), CASP10 (2012), CASP11 (2014), and CASP12 (2016).

In short, the method first compares the proposed sequence with the ones in protein databases to identify similar structural templates and align its amino acid sequences. Next, the unaligned sequences are built by *ab initio* folding and a simulation of different assemblies with the aligned and unaligned sequences is made by Monte Carlo simulations, creating a set of possible candidates. Then, a selection of the lowest free-energy conformations is made and, starting from this model, a second round of assembly simulation is performed in order to refine the global topology [45].

To evaluate the goodness of the predictions, in addition to the TM-Score and the residual RMSD present in the literature, Zhang Lab—University of Michigan has defined a parameter called C-Score. When it is used to evaluate the structural properties, C is typically in the range of [-5,2], where a higher value implies higher confidence in the structure prediction, and models with a C-Score greater than -1.5 are considered reliable predictions.

**Fig 3:**
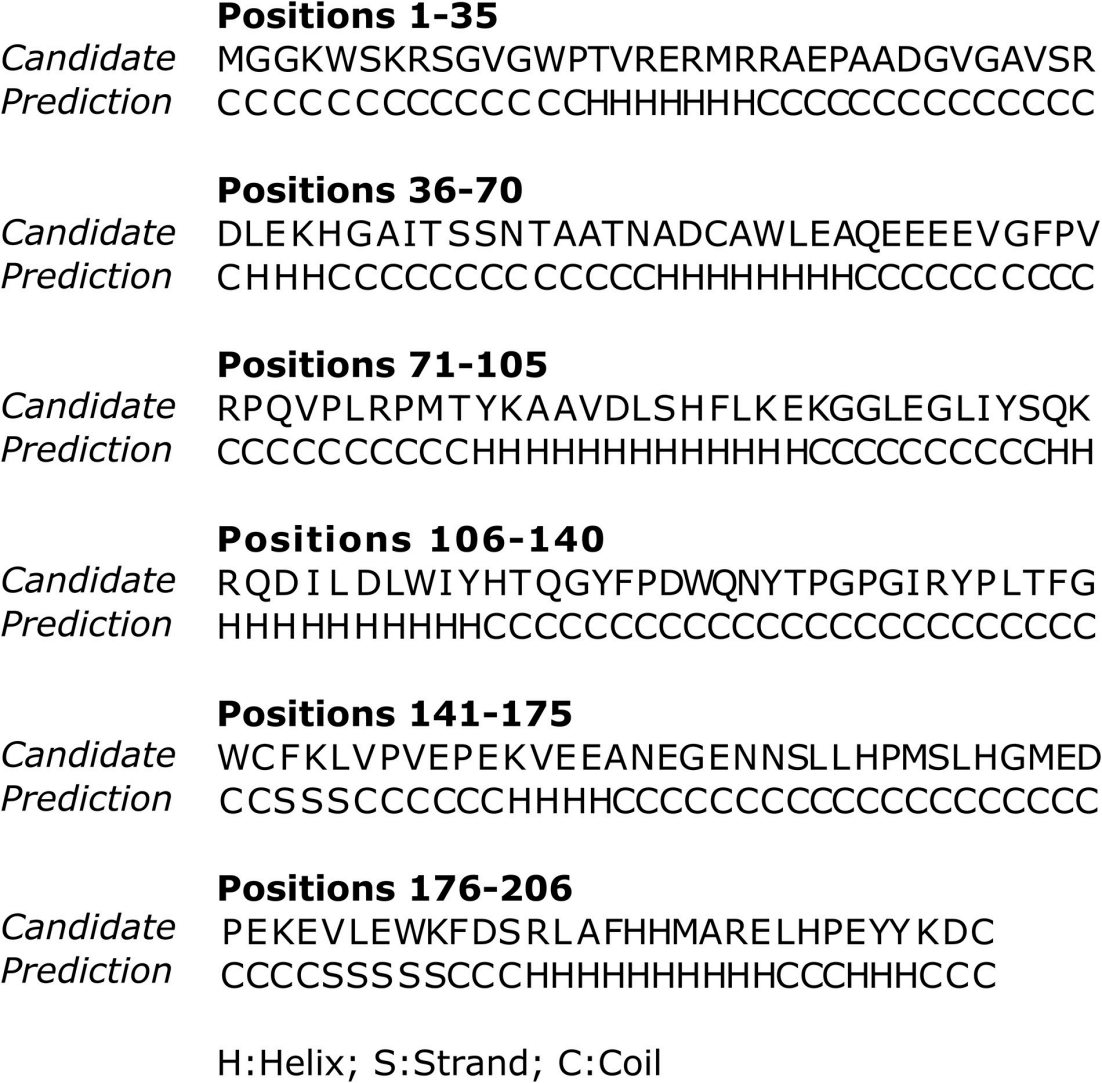
Prediction of the secondary structure of the candidate sequence by I-TASSER. In this graph we represent the amino acid sequence of our candidate, and below, the secondary structure associated to each AA predicted with I-TASSER. Here, H indicates Helix, S Strand, and C Coil.

We analyzed the secondary structure of the candidate sequence. In Fig 3, the secondary structure predicted with I-TASSER for the candidate sequence is displayed. To show the plausibility of the predicted secondary structure, we emphasize that in sequence 2XI1 of Protein Data Bank, which is based in the work by Singh et al. [46], a secondary structure for most part of the C-terminal highly conserved domain of HIV1-Nef is showed, in which there is a high level of agreement with our prediction. Residues 149-178 are disordered in the crystal structure obtained in [46], and hence in that region the sequence is recorded but no coordinates are determined. In 2XI1 the following substructures appear:

alpha helix: 83–95
alpha helix: 106–120
beta strand: 145–149
beta strand: 183–187
3/10 helix: 189–192
alpha helix: 196–200,

which are in good agreement with the structure predicted by I-TASSER.

**Fig 4:**
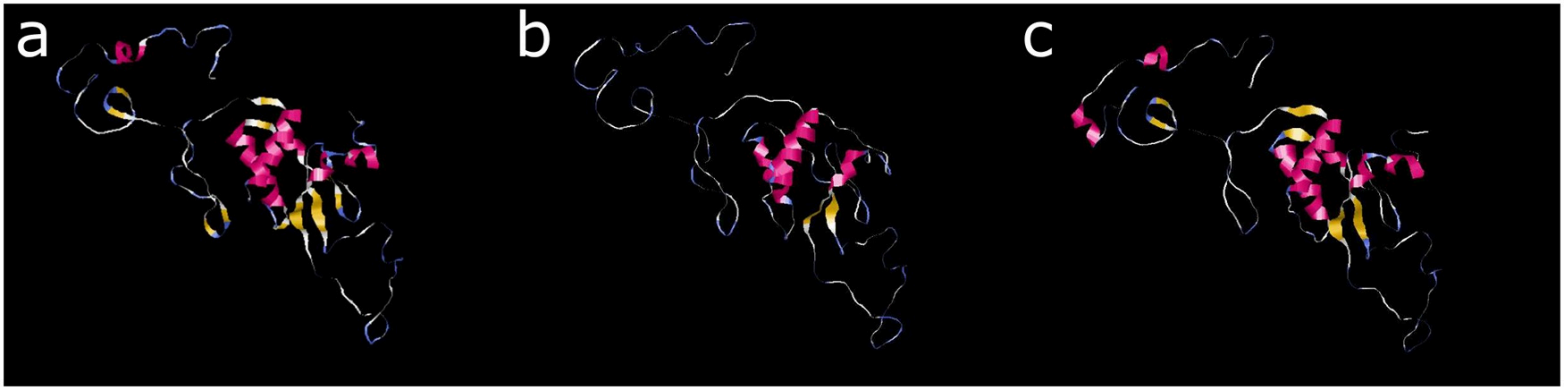
Tertiary structures by I-tasser and Phyre-2. In this molecular graph we illustrate the resemblance between the tertiary structure of (a) the candidate with I-Tasser, (b) the candidate with Phyre-2, and (c) the sequence 2XI1 with Phyre-2.

We also analyzed the tertiary structure of the candidate sequence, which is represented graphically in Fig 4, panel (a). The prediction obtained for our candidate is highly reliable, since the C-Score of the model is 1.42, and the cutoff value to consider a good prediction is -1.5. Besides, the predicted structure is very similar to the one observed in the Nef protein 3TB8 of Protein Data Bank. This similarity achieved a TM-score of 0.896 in I-TASSER. The TM-score scales the structural similarity between two protein structures. The TM-score ranges on a scale from 0 to 1, with 1 denoting a perfect match and where a scoring greater than 0.5 means that it assumes generally the same fold [47].

In addition to the analysis done with I-TASSER, we have used Phyre2 [48] web portal for protein folding to estimate the structure of the candidate. In Fig 4, panel (b) we illustrate the tertiary structure obtained by Phyre2. It can be observed that the folding is very similar to the one obtained by I-TASSER, depicted in Fig 4, panel (a). Moreover, results of Phyre2 indicate that 98% of the residues were modeled with a confidence >90%, using as model the 3TB8 protein, which is the same that I-TASSER used as model for its predictions. Therefore, in this case, both predictions coincide, which reinforces the likelihood that the candidate will fold as predicted.

Additionally, we have studied the sequence 2XI1 with Phyre2. As in the prediction of the candidate with Phyre2, the main model to estimate the tertiary structure of 2XI1 is the protein 3TB8, with 94% of the residues modeled with a confidence >90% using this template. In Fig 4, panel (c) we illustrate the predicted folding of the sequence 2XI1 by Phyre2, which is very similar to the one obtained with the candidate by using Phyre2, and even more similar to the folding obtained by I-TASSER.

Finally, we can see that the folding predictions done with I-TASSER and Phyre2 were based in the same protein (3TB8) and were very similar (see Fig 4).

We did also a BLAST [49] search of the candidate sequence, and we obtained that the five most similar sequences to the candidate sequence were the following ones:

1. **AAX86040.1**, with a total score of 420 and an identity of 97%. It corresponds to a synthetic construct of a HIV-1 Clade B consensus Nef protein presented in [50], where Kavanagh et al. transfected antigen-presenting cells (APCs) with mRNA encoding Gag-p24 and cytoplasmic, lysosomal, and secreted forms of Nef. They found that transfection of APCs with a Nef construct bearing lysosomal targeting sygnals produced rapid and prolongued antigen presentation to CD4^+^ and CD8^+^ T cells [50].
2. **AAX39503.1**, with a total score of 418 and an identity of 97%. It corresponds to a synthetic construct of a consensus Nef protein, which was used in [51], along with other sequences, to validate the FATT-CTL assay, which is a novel method for the measurement of CTL-mediated cytotoxicity.
3. **AAA87523.1**, with a total score of 416 and an identity of 95% and **AAA87527.1**, with a total score of 415 and an identity of 94%. They corresponds to 2 of the 88 sequences of Nef protein of HIV-I, analyzed by Michael et al. in [52].
4. **AAA63871.1**, with a total score of 414 and an identity of 94%. It corresponds to 1 of the 90 sequences of a Nef protein of HIV-I, analyzed by Huang, Zhang, and Ho in [53].

**Fig 5:**
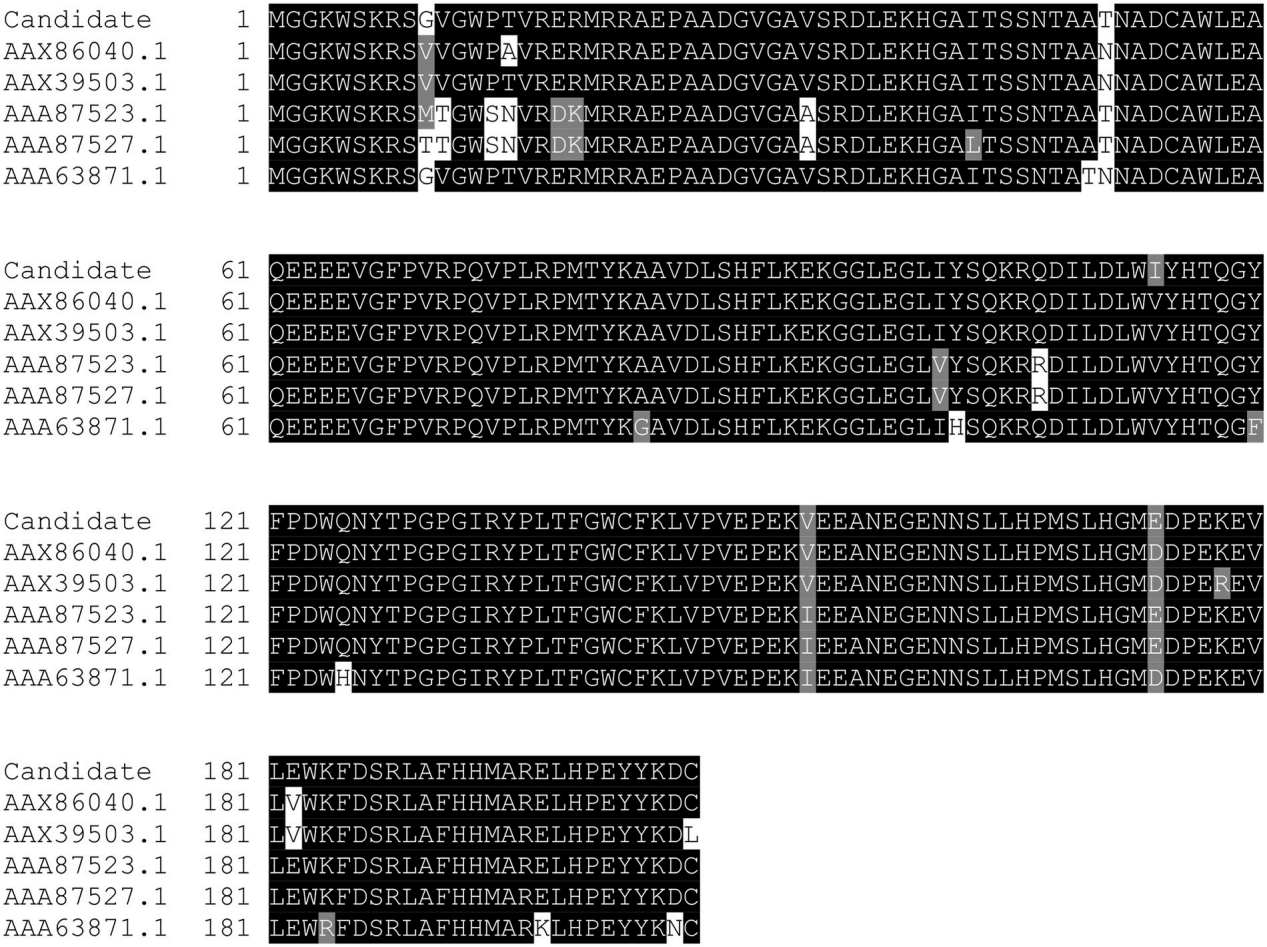
BLAST alignment of the candidate with AAX86040.1, AAX39503.1, AAA87523.1, AAA87527.1, and AAA63871.1. In this graph we depict the alignments of the candidate with the five sequences for the BLAST analysis (AAX86040.1, AAX39503.1, AAA87523.1, AAA87527.1, and AAA63871.1). When the residues were identical, they were shaded in black; if they were not identical but at least similar, they were colored in grey; finally, when there were no similarities among residuals, they were shaded in white.

In Fig 5, we depict the alignments of the candidate with the five sequences for the BLAST analysis. When the residues were identical, they were shaded in black; if they were not identical but at least similar, they were colored in grey; finally, when there were no similarities among residuals, they were shaded in white.

In addition, we used VaxiJen [54], which is a server for alignment-independent prediction of protective antigens. It uses bacterial, viral and tumour protein datasets to derive models for prediction of whole protein antigenicity. With our candidate sequence the overall prediction for the antigen obtained with VaxiJen selecting “Virus” as target organism was 0.6895 (Probable antigen). The threshold value to be considered probable antigen was 0.4. For more information about VaxiJen, we refer the reader to [55].

The overall predictions obtained in VaxiJen for the 5 strings closer to our candidate sequence in the BLAST search were:

0.6380 for AAX86040.1
0.6409 for AAX39503.1
0.6688 for AAA87523.1
0.6747 for AAA87527.1
0.6599 for AAA63871.1

Next, we have compared our candidate with other sequences obtained by three different algorithms. The first is one of the candidates obtained with the unweighted algorithm in our previous work [10] (we selected among our solutions the candidate with the number of amino acids closest to 206, i.e., closest to the length established for Nef [42], but without exceeding this number); the second was obtained by using LANL’s Epigraph [56]; and the third was a consensus sequence obtained by LANL’s Consensus [57].

In Table 4, the resultant estimated Class I immunogenicity [40] and mismatch proportion for the four strings can be found. As expected, the estimated immunogenicity value of our weighted candidate was better than the ones of the other three, suggesting that it would generate a more immunogenic response. The mismatch proportion was very similar (near to 0.511) between the weighted, epigraph, and consensus candidates. This result was expected, since we chose a candidate with high alignment, which implies a smaller number of mismatches, and both epigraph and consensus methods are expected to resemble the natural proteins [56, 57]. Finally, since the unweighted candidate did not take into account the alignment, it scored a very high mismatch ratio (equal to 1).

**Table 4:** Comparison between the weighted, unweighted, epigraph, and consensus candidates.

	Class I immunogenicity	mismatch average
Weighted	1.8685	0.5115
Unweighted	1.8409	1
Epigraph	1.2307	0.5114
Consensus	1.4103	0.5109

For the purpose of comparison, we have used also a hill-climbing algorithm, as we did in [10]. In this case we used a multi-objective hill climbing algorithm analogous to the one described in [58]. As we did in the Materials and methods section, we considered sequences *u* of target strings and the corresponding phenotypes *o*(*u*) obtained by taking the overlapping sum of the strings in *u*. We selected randomly 10 sequences $h_{i_{1}},\ldots ,h_{i_{10}}$ from the set of host strings and the corresponding sequences $u_{i_{1}},\ldots ,u_{i_{10}}$ of target strings, and for each sequence $u_{i_{j}}$ we performed the following procedure:

First, we initialized to $\left\{u_{i_{j}}\right\}$ the set *ND*_*i*_ of non-dominated solutions. Then, we tried to simulate mutations sequentially in positions of the sequences in *ND*_*i*_, by replacing a target string by another target string at the same position in some of the host strings *h*_1_, …, *h*_169_. If at some point we get a sequence *u*′ non-dominated for some sequence in *ND*_*i*_, then we add the sequence *u*′ to the set *ND*_*i*_ and we repeat the process from the beginning. Instead of repeating this process until no new non-dominated sequence is found, due to the excessive time to required to attain this, we simulated a total of 10^6^ mutations.

**Fig 6:**
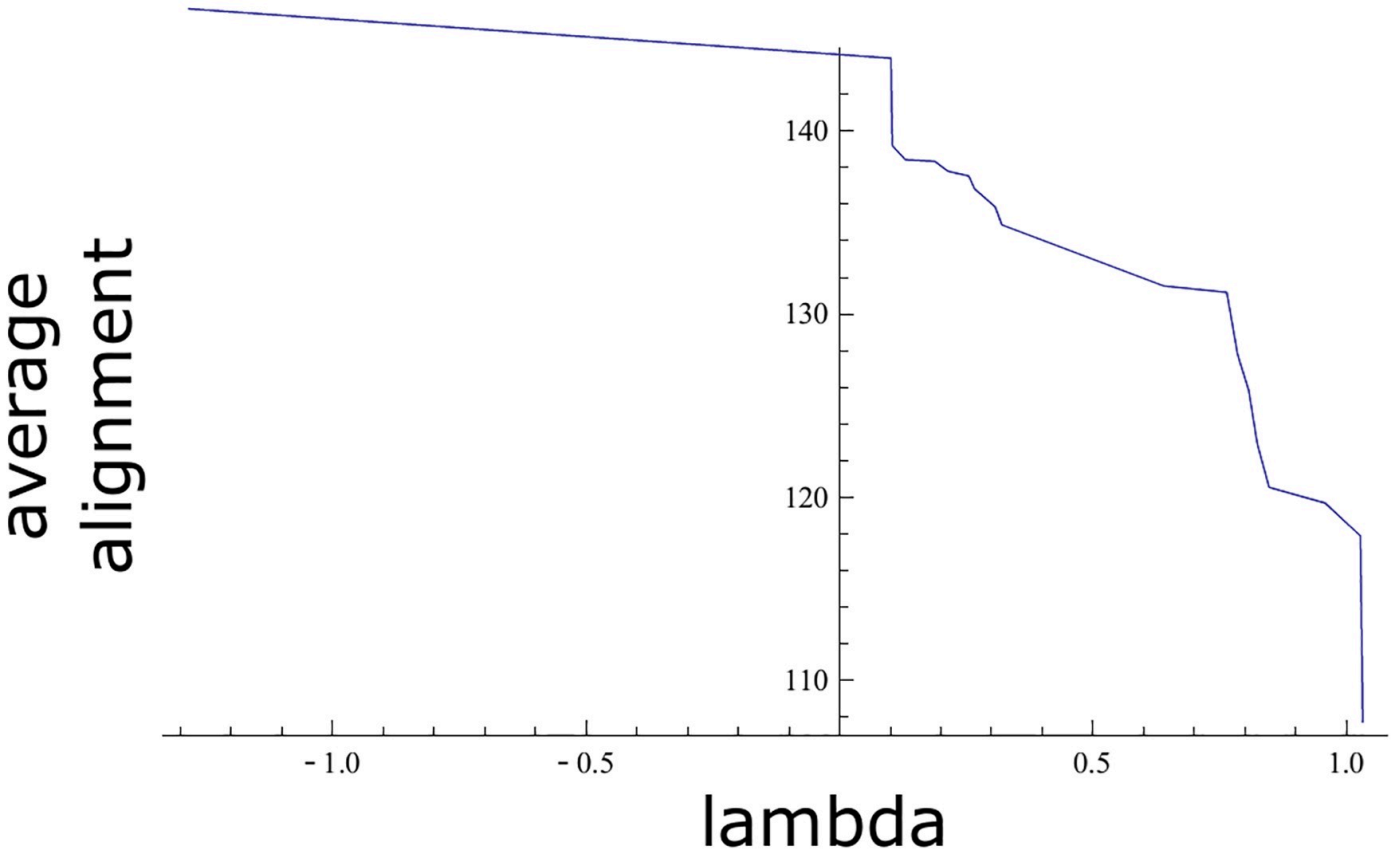
Estimation of the Pareto front in the hill-climbing algorithm. The line represents the non-dominated solutions found with the multi-objective hill climbing algorithm. The X axis indicates the λ value, while the Y axis indicates the alignment score.

We took the union of the non-dominated sets *ND*_1_, …, *ND*_10_ and selected the phenotypes of the non-dominated elements in this union as an approximation to the true Pareto front, which is shown in Fig 6 and in Table 5. The approximation to the Pareto front is worse than the one shown in Fig 2, obtained by using the genetic algorithm, in the sense that every solution shown in Fig 6 is dominated by at least one solution in Fig 2.

**Table 5:** Numerical values for the estimation of the Pareto front in the hill-climbing algorithm.

(-1.2852,146.68)
(0.10136,143.982)
(0.10365,139.213)
(0.12965,138.432)
(0.18779,138.349)
(0.21379,137.811)
(0.25488,137.55)
(0.26566,136.87)
(0.30675,135.87)
(0.32028,134.876)
(0.63875,131.544)
(0.76386,131.183)
(0.78531,127.781)
(0.80699,125.817)
(0.82376,122.917)
(0.84713,120.538)
(0.959,119.663)
(1.02748,117.893)
(1.03195,107.686)

## Discussion

In this paper, we generalized the notion of λ-superstrings from [10] to the weighted case. We developed an exact algorithm for a corresponding combinatorial optimization problem based on integer programming, extending the model from the previous paper by introducing a weight function on the target strings (which can take both positive and negative values). We consider that weighted λ-superstring criterion could be useful to fight the high mutability and escape mutations of viruses like HIV, HCV, or Influenza, since it gives a balanced protection against all the variants considered, by ensuring that at the overall the immunogenicity of the epitopes in each variant is at least λ. We also described a model taking into account good pairwise alignments of the obtained superstring with the host strings, and presented a heuristic approach based on a multi-objective genetic algorithm. By considering the alignment as a target to optimize by our algorithm, the weighted λ-superstrings obtained by using the genetic algorithm correspond to pseudoproteins structurally similar to the original ones taken from the patients, instead of being just epitope aggregates, opening the doors to possible improvements in the current methodology of epitope vaccine design.

In order to evaluate the performance of our algorithm, first, we analyzed the estimation of the Pareto front obtained with a multi-objective hill-climbing algorithm, which gave worse solutions than the one obtained by the genetic algorithm. Then, we selected a vaccine candidate from the Pareto front and studied its effectiveness *in silico*. Due to the weighted λ-superstring condition, and the positive λ value, this pseudo-protein would likely protect against all virus variants considered. Besides, VaxiJen analysis corroborated that the vaccine would be a probable antigen. Next, the structure and resemblance to the native protein were evaluated by several bioinformatic tools (such as Blast, Phyre 2 or I-Tasser), which indicated that our candidate was very similar to HIV-1 2XI1 and 3TB8 sequences. Then, we performed a comparison among our weighted candidate, one of the candidates obtained with the unweighted algorithm in our previous work [10], a candidate obtained by using LANL’s Epigraph, and a consensus sequence. In this analysis, we observed that the mismatch proportion was worse in the unweighted candidate, which was expected, since the algorithm in [10] did not optimize the alignment. Besides, the estimated Class I immunogenicity [40] of the weighted candidate was bigger than the estimated immunogenicity for the candidates found with other methods, suggesting that it would generate a more immunogenic response.

Additionally, in order to study the sensitivity of the method, we have also analyzed D and G HIV subtypes, and they yielded similar results, indicating that the method is robust. These analyses can be found in S2 Appendix.

An important point of future work on weighted λ-superstrings is to determine the extent of practical applicability of the presented models and algorithms to vaccine design, in particular to assess the immunological value of the resulting candidate vaccines. In this regard, we have recently described a functional method to decipher T-cell epitopes of the bacterial and human pathogen *Listeria monocytogenes (Listeria)* based on combination of bioinformatics predictions of epitopes binding to MHC molecules and functional assays [59]. Our hypothesis was based in the use of two *Listeria* antigens, the listeriolysin O (LLO) and the glyceraldehyde-3-phosphate-dehydrogenase (GAPDH) that elicits strong CD4+ and CD8+ T cell responses [60], [61]. Our method to test vaccine candidates was based in the use of predicted peptides from the bioinformatics analysis to activate dendritic cells *in vitro* and elicit high delayed T hypersensitivity (DTH) responses *in vivo*, combined to measurements of IL-12 production as the cytokine that best correlates with immune protection.

In order to adapt the methodology just described to the framework of vaccine design using weighted λ-superstrings, we will use in future work the full-length sequence of LLO for the thirteen recognized serotypes of *Listeria Monocytogenes* to design B and T-cell epitope vaccines applying weigthed λ-superstrings that gather the genetic diversity of the pathogen by means of the consideration of the different serotypes, and we will compare the epitopes obtained with those of previous studies. Next, we will use the weighted λ-superstrings obtained with the selected epitopes in our functional method of vaccine candidates testing. Finally, our success in predicting efficient LLO epitopes for vaccination and the construction of the subsequent λ-superstrings will be relevant for other intracellular bacteria for which we currently lack available vaccines, such as *Mycobacterium tuberculosis, Salmonella enteritidis*, or *Chlamydia trachomatis*, among others.

One of the lines considered as future work is to evaluate if the algorithm gives better results when we consider near-matches of the epitopes instead of exact matches, by changing the fitness function. By this approach, we would obtain vaccine candidates that induce cross-reactive T-Cells, which could be activated during the infection of an unrelated heterologous virus. Cross-reaction and its benefits have been widely observed in several infections [62, 63], and since their positive effects in vaccination is promising [64, 65], we consider that this approach might enhance the effectiveness of our method.

Moreover, we would like to consider, besides the weights corresponding to immunogenicities, other kinds of weights at the same time, addressing different biologically motivated goals with different weights. For example, one could consider weights associated to the relative frequencies of the epitopes.

In summary, here we have presented two algorithms for computational vaccine design. Our results indicate that with this methodology, we can obtain weighted λ-superstrings that resemble native protein structures, and protect well-balancedly against the whole group of considered virus variants, minimizing the chances of perpetuating the infection due to escape mutations.

## Supporting information

S1 Appendix Mathematical proof. (PDF)

S2 Appendix Additional sub-analyses. (PDF)

S1 Fig Distribution of the values in the Pareto front. Histograms representing the frequencies of the λ (a) and alignment (b) values of the estimation of the Pareto front obtained with the genetic algorithm. The Y axis represents the frequency, while the X axis indicates the λ value (a) and the alignment score (b).
(TIF)

S1 Table GenBank IDs of the sequences for the Nef protein. (PDF)

S2 Table Experimental values of the immunogenicities of the epitopes. (PDF)

S3 Table Positions and sequences of the conserved regions for the Nef protein. (PDF)
