# Supplementary material for: Weighted lambda superstrings applied to vaccine design
Source: PLoS One. 2019 Feb 8;14(2):e0211714. doi: 10.1371/journal.pone.0211714 (PMC6368308; doi:10.1371/journal.pone.0211714)
Supplement: S1 Appendix — (PDF) [file pone.0211714.s001.pdf]

S1 Appendix: Proof of Proposition 5

Proposition 5 (Restated)**.** *Let $(\mathcal{C}, w, \lambda)$ be an instance to the* Shortest Weighted λ-Cover Superstring *problem, and let G be its derived distance graph. Then, there exists a weighted λ-cover superstring for $(\mathcal{C}, w)$ of length at most ℓ if and only if G contains a w-feasible directed cycle C of cost at most ℓ.*

Proof. First, suppose that $\mathbf{v}$ is a weighted λ-cover superstring for $(\mathcal{C}, w)$ of length at most ℓ. For every $X \in \mathcal{C}$, let $X_\mathbf{v} \subseteq X$ denote the set of strings in X that are substrings of $\mathbf{v}$. Then, we have $\sum_{\mathbf{t} \in X_\mathbf{v}} w(\mathbf{t}) \geq \lambda$. Let Z denote the set of maximal elements of the set $Y := \bigcup_{X \in \mathcal{C}} X_\mathbf{v}$ partially ordered with respect to the substring relation, that is,

$$Z = \{\mathbf{s} \in Y \,:\, (\forall \mathbf{y} \in Y)(\text{if } \mathbf{s} \subseteq \mathbf{y} \text{ then } \mathbf{s} = \mathbf{y})\}\,.$$

Let us order the elements of Z as $(\mathbf{z}_1, \ldots, \mathbf{z}_p)$ according to the order of their first appearance as substrings of $\mathbf{v}$. Since no string in Z is a substring of another string in Z, this ordering is well defined and unique. Note that $(\mathbf{z}_1, \ldots, \mathbf{z}_p)$ defines a directed path in $G - s^*$. Extend this path with s^* to a cycle $C = (\mathbf{z}_1, \ldots, \mathbf{z}_p, s^*)$ in G. We claim that C is a w-feasible cycle of cost at most $\ell(\mathbf{v})$. By the definition of the distance graph, the cost of C is equal to

$$\sum_{i=1}^{p} c(\mathbf{z}_i, \mathbf{z}_{i+1}) + c(\mathbf{z}_p, s^*) + c(s^*, \mathbf{z}_1) = \sum_{i=1}^{p} (\ell(\mathbf{z}_i) - ov(\mathbf{z}_i, \mathbf{z}_{i+1})) + \ell(\mathbf{z}_p)\,,$$

that is, the length of the overlapping sum of strings $\mathbf{z}_1, \ldots, \mathbf{z}_p$ in this order. This is not more than the total number of characters of $\mathbf{v}$ appearing in the first occurrence of some $\mathbf{z}_i$ as a substring of $\mathbf{v}$, which clearly does not exceed the length of $\mathbf{v}$. The defining properties of w-feasible cycles can also be easily verified: first, we have $s^* \in V(C)$ by construction. Second, every two distinct vertices $\mathbf{s}, \mathbf{t} \in V(C) \cap T$ are from Z, and hence neither of $\mathbf{s}$ and $\mathbf{t}$ is a substring of the other string. Finally, let $X \in \mathcal{C}$, and consider the set $X_C \subseteq X$ of strings in X covered by C. By definition of C, the set X_C equals the set of all strings in X that are substrings of some string in Z, which, in turn, equals to the set of strings in X that are substrings of $\mathbf{v}$. Hence, $X_C = X_\mathbf{v}$, and the covering requirement $\sum_{\mathbf{t} \in X_C} w(\mathbf{t}) \geq \lambda$ for C follows from the corresponding requirement for $\mathbf{v}$.

Conversely, suppose that $C = (\mathbf{z}_1, \ldots, \mathbf{z}_p, s^*)$ is a w-feasible directed cycle in G of cost at most ℓ. Let $\mathbf{v}$ be the string defined as the overlapping sum of strings $\mathbf{z}_1, \ldots, \mathbf{z}_p$ in this order. The definition of the distance graph implies that the length of $\mathbf{v}$ is equal to the cost of C, and hence at most ℓ. Also, the fact that C is w-feasible implies that $\mathbf{v}$ is a weighted λ-cover superstring for $(\mathcal{C}, w)$. Indeed, for every $X \in \mathcal{C}$, if $X_\mathbf{v}$ denotes the set of strings in X that are substrings of $\mathbf{v}$, then $X_C \subseteq X_\mathbf{v}$, and consequently $\sum_{\mathbf{t} \in X_\mathbf{v}} w(\mathbf{t}) \geq \sum_{\mathbf{t} \in X_C} w(\mathbf{t}) \geq \lambda$. This completes the proof. □
