# Supplementary material for: Weighted lambda superstrings applied to vaccine design
Source: PLoS One. 2019 Feb 8;14(2):e0211714. doi: 10.1371/journal.pone.0211714 (PMC6368308; doi:10.1371/journal.pone.0211714)
Supplement: S2 Appendix — (PDF) [file pone.0211714.s002.pdf]

S2 Appendix: Additional sub-studies

Additionally, in order to study the sensitivity of the method, we have also analyzed D and G HIV subtypes, and they yielded similar results, indicating that the method is robust. Here, we summarize the results of those sub-analyses to illustrate that different subtypes of the virus seem to achieve similar results both in immunogenicity and structure.

The first sub-analysis was performed over all the host strings of the HIV-1 subtype D found in Los Alamos HIV Database (N=474). After removing repeated and problematic sequences (those with ambiguous amino acids or nonsense codons), the size of the host string set was reduced to 266. Next, we estimated computationally the immunogenicity with the IEDB tool, and applied our genetic algorithm in order to obtain the estimation of the Pareto front. The values for this sub study ranged between (-1.26, 159.2) and (4.37, -93.31), being the first component the λ value and the second one the alignment scoring. Then, we selected the following candidate:

MGGKWSKSSIVGWPAIRERIRRTDPAAEGVGAASRDLERHGAITSSNTAQTNPDCAWLEAQEED
EEVGFPVRPQVPLRPMTYKHLIATFRMTYKGALDLSHFLKEKGGLEGLIWSQKRQEILDLWVYHTQ
GYFPDWQNYTPGPGIRYPLTFGWCFELVPVDPKEVEEDTEGENNCLLHPMCQHGMEDPEREVLKW
RFNSRLAFEHKARVLHPEFYKDC,

which presented a good balance between λ value (1.08) and alignment (148.36), and we studied its predicted structure with I-TASSER. The results indicated that, once again, the candidate was very similar to the 3TB8 protein, with a TM-Score of 0.906, which suggests that it will very likely have a stable structure. Besides, the predicted model (represented in Figure S2.1) was considered highly reliable, since the C-Score obtained was of 1.00.

Figure S2. 1: **I-TASSER prediction of the candidate for the D subtype.**

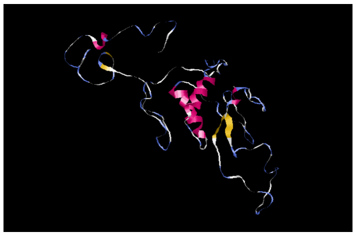

We analyzed the obtained string with VaxiJen selecting "Virus" as target organism, and we obtained an overall prediction of 0.6922 (Probable antigen). The threshold value indicated in VaxiJen to be considered probable antigen was 0.4.

The second sub-analysis was carried out over all the host strings of the HIV-1 subtype G found in Los Alamos HIV Database (N=190). After removing repeated and problematic sequences, the size of the host string set was reduced to 151. Then, we proceeded as in the previous analyses and obtained the estimation of the Pareto front, which in this case ranged between (-1.96, 160.47) and (5.37, -145.4). For this set, we selected the following candidate:

MGGKWSKCSIVGWPQVRERIRRTPPAPAAEGVGAASQDLARHGAITSSNTVTNNPDCAWLEAQE
EDSEVGFPVRPQVPVRPMTYKAAFDLSFFLKEKGGLDGLIYSKQRQDILDLWVYNTQGFFPDWQNY
TPGPGTRFPLTFGWCFKLEPMDPAEVEEANKGENNSLLHPICQHGMEDEDREVLVWRFDSSLARRH
IARELHPEYYKDC,

which presented a good balance between λ value (2.24) and alignment (155.1), and we studied its predicted structure with I-TASSER. The candidate was very similar to the 3TB8 protein, with a TM-Score of 0.937, indicating once again that it would probably present a stable structure. Moreover, the predicted model (represented in Figure S2.2) was considered highly reliable, since the C-Score obtained was of 1.71.

We analyzed also the obtained string with VaxiJen, and we obtained an overall prediction of 0.5994 (Probable antigen).

Figure S2. 2: **I-TASSER prediction of the candidate for the G subtype.**

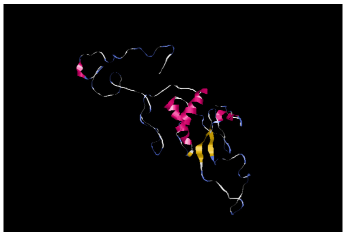

Since the results were very similar, the analyses suggested that our method is robust.
