# Supplementary figures and images for: Weighted lambda superstrings applied to vaccine design
Source: PLoS One. 2019 Feb 8;14(2):e0211714. doi: 10.1371/journal.pone.0211714 (PMC6368308; doi:10.1371/journal.pone.0211714)

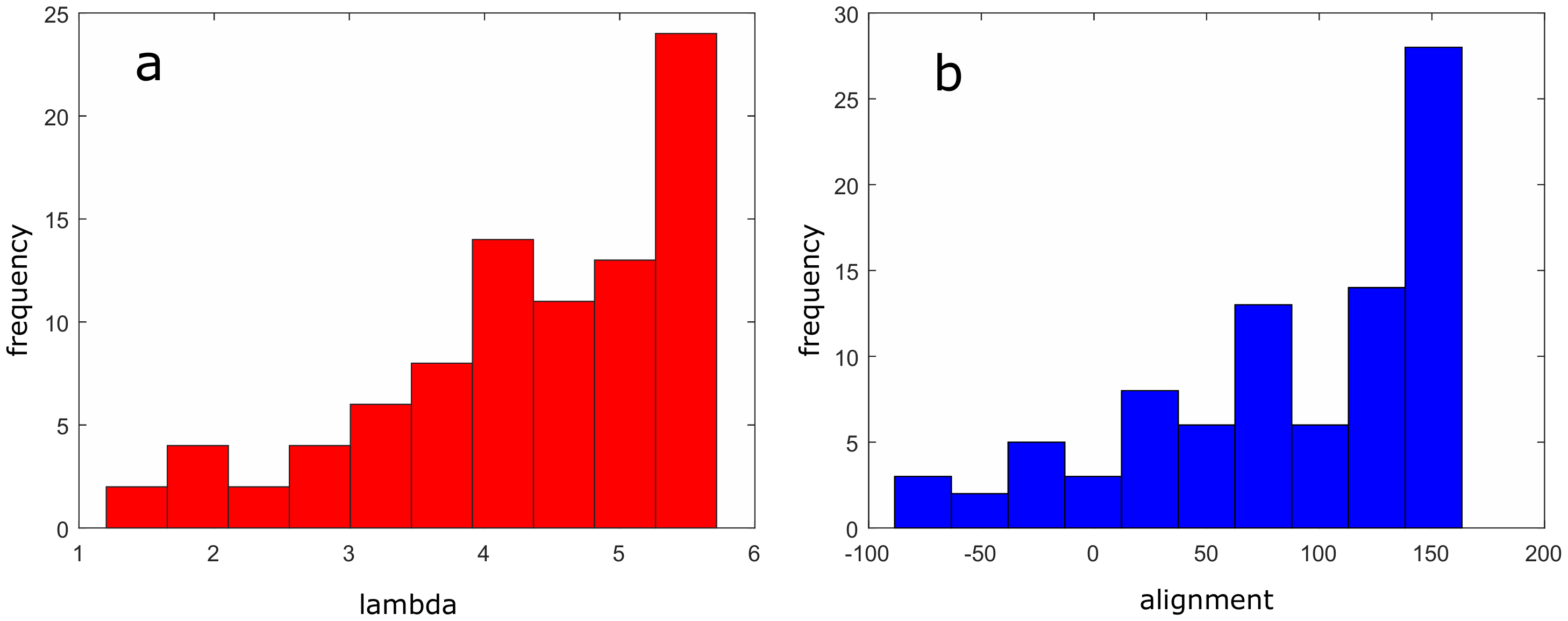

Supplement: S1 Fig — Histograms representing the frequencies of the λ (a) and alignment (b) values of the estimation of the Pareto front obtained with the genetic algorithm. The Y axis represents the frequency, while the X axis indicates the λ value (a) and the alignment score (b). (TIF) [file pone.0211714.s003.tif]
