# Supplementary material for: Weighted lambda superstrings applied to vaccine design
Source: PLoS One. 2019 Feb 8;14(2):e0211714. doi: 10.1371/journal.pone.0211714 (PMC6368308; doi:10.1371/journal.pone.0211714)
Supplement: S1 Table — (PDF) [file pone.0211714.s004.pdf]

Table S1: GenBank IDs of the sequences for the Nef protein

AB012824	AF129350	AF203161	AF538305	AY835753	M58173
AB034257	AF129351	AF203165	AF538306	AY835762	M93259
AB034272	AF129352	AF203172	AJ271445	AY835765	U03295
AB078005	AF129354	AF203180	AJ430664	AY835770	U03338
AB221005	AF129355	AF203188	AY037269	AY835772	U03343
AF004394	AF129362	AF203192	AY037282	AY835776	U12055
AF011471	AF129364	AF203194	AY116676	AY835779	U16863
AF011474	AF129369	AF203198	AY116713	AY835780	U16875
AF011481	AF129370	AF219672	AY116714	AY857022	U16934
AF011487	AF129372	AF219685	AY116727	AY857144	U23487
AF011493	AF129373	AF219691	AY116781	AY899356	U24455
AF042101	AF129375	AF219729	AY116805	AY899382	U26087
AF047082	AF129376	AF219755	AY116830	DQ007902	U26110
AF063926	AF129377	AF219760	AY121441	DQ085869	U26119
AF069139	AF129378	AF219765	AY173951	DQ121815	U26138
AF120745	AF129379	AF219771	AY308762	DQ121883	U34603
AF120772	AF129382	AF219782	AY314063	DQ127537	U43106
AF120840	AF129388	AF219792	AY331285	DQ127548	U44444
AF120851	AF129389	AF219800	AY331290	DQ487191	U44450
AF120867	AF129390	AF219812	AY331293	DQ659737	U44462
AF120887	AF129392	AF219819	AY352275	L07422	U44468
AF120898	AF129394	AF219845	AY444311	L15482	U66543
AF120909	AF203108	AF238268	AY713408	L15489	U69584
AF129334	AF203111	AF252897	AY739040	L15500	U71182
AF129335	AF203116	AF252910	AY779550	L15515	
AF129342	AF203126	AF462708	AY786630	L15518	
AF129343	AF203137	AF462753	AY786750	M17451	
AF129346	AF203141	AF538302	AY835748	M21098	
AF129347	AF203153	AF538304	AY835751	M26727	
