# Supplementary material for: Weighted lambda superstrings applied to vaccine design
Source: PLoS One. 2019 Feb 8;14(2):e0211714. doi: 10.1371/journal.pone.0211714 (PMC6368308; doi:10.1371/journal.pone.0211714)
Supplement: S2 Table — (PDF) [file pone.0211714.s005.pdf]

Table S2: Experimental values of the immunogenicities of the epitopes

AAVDLSHFL	0	LTFGWCFKL	1
AFHHVAREL	1	LTFGWCFKLV	1
AVDLSHFL	0	PLTFGWCYKL	0
AVDLSHFLK	0,57	QEILDLWVY	0,63
EWRFDSRL	1	QVPLRPMTYK	0,68
FPDWQNYT	0	RPMTYKAAL	0,41
FPVRPQVPL	0,94	RPQVPLRPM	1
FPVTPQVPL	0,31	RYPLTFGWCF	1
KAAVDLSHFL	1	TPGPGIRYPL	1
KEKGGLEGL	0,5	TPGPGVRYPL	1
KRQEILDLWVY	1	TQGYFPDWQNY	1
VLEWRFDSRL	0,2	VPLRPMTY	1
YPLTFGWCF	1	DLSHFLKEKGGLEGL	0,5
HHVARELHPEYFKNC	1	RLAFHHVARELHPE	1
EWRFDSRLAFHHVAREL	1	GVRYPLTFGWCYKLVP	1
PEKEVLVWKFDSRLAFHH	1	YKAAVDLSHFLKEKGGL	0,75
