# Supplementary material for: Weighted lambda superstrings applied to vaccine design
Source: PLoS One. 2019 Feb 8;14(2):e0211714. doi: 10.1371/journal.pone.0211714 (PMC6368308; doi:10.1371/journal.pone.0211714)
Supplement: S3 Table — (PDF) [file pone.0211714.s006.pdf]

Table S3: Positions and sequences of the conserved regions for the Nef protein

1	MGGKWSK	115	Y
12	GW	117	TQG
17	RERM	121	FPDWQNYTPGPG
24	EPAA	134	R
29	GVGA	136	PLTFGWCFKLVPV
34	SRDL	150	P
41	GA	154	E
44	T	156	A
46	SNT	159	GEN
52	NA	164	LLHP
55	CAWLEAQE	171	HG
64	EEVGFPV	175	D
72	PQVPLRPMT	177	E
82	K	179	EVL
84	A	183	W
86	DLSHFL	185	FDS
93	EKGGLEGL	189	LAF
103	SQ	193	H
106	RQ	195	ARE
109	ILDLW	199	HPEYYK
